# Supplementary material for: The interactome of intact mitochondria by cross-linking mass spectrometry provides evidence for coexisting respiratory supercomplexes
Source: Mol Cell Proteomics. 2017 Dec 8;17(2):216–32. doi: 10.1074/mcp.RA117.000470 (PMC5795388; doi:10.1074/mcp.RA117.000470)
Supplement: Supplemental Data [file supp_RA117.000470_133922_0_supp_23978_fzffwf.zip › spectra_annotation/mito_spectra_annotation/236-1-4-1-12-1.pdf]

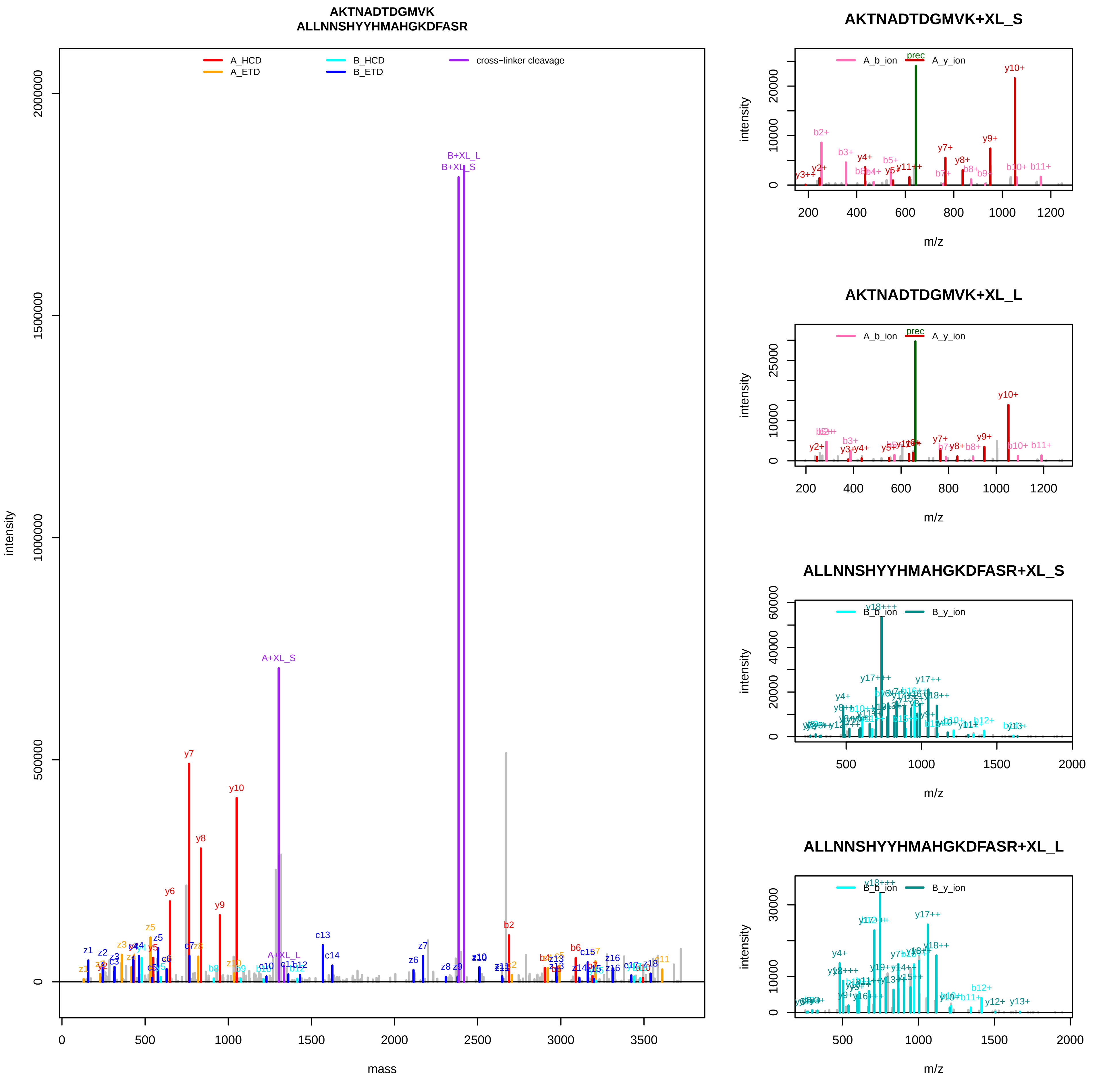

AKTNADTDGMVK
ALLNNSHYYHMAHGKDFASR
A_HCD
A_ETD
B_HCD
B_ETD
cross−linker cleavage
B+XL_L
B+XL_S
A+XL_S
A+XL_L
y7
y10
y8
y6
y9
b2
c13
c14
z7
z6
z8
z9
z10
z11
z5
c15
z16
z18
b6
intensity
mass
0
500000
1000000
1500000
2000000
0
500
1000
1500
2000
2500
3000
3500
AKTNADTDGMVK+XL_S
AKTNADTDGMVK+XL_L
ALLNNSHYYHMAHGKDFASR+XL_S
ALLNNSHYYHMAHGKDFASR+XL_L
A_b_ion
A_y_ion
B_b_ion
B_y_ion
prec
y10+
y9+
y8+
y7+
b2+
b3+
y4+
b10+
b11+
y18+++
y17+++
y17++
y18++
y4+
b12+
y13+
intensity
m/z
